# Cell-free stem cell-derived extract formulation for treatment of knee osteoarthritis: study protocol for a preliminary non-randomized, open-label, multi-center feasibility and safety study

**DOI:** 10.1186/s13018-021-02672-3

**Published:** 2021-08-20

**Authors:** Ashim Gupta, Nicola Maffulli, Hugo C. Rodriguez, R. Justin Mistovich, Kristin Delfino, Craig Cady, Anne-Marie Fauser, Echo D. Cundiff, Marte A. Martinez, Anish G. Potty

**Affiliations:** 1 General Therapeutics, 2956 Washington Blvd, Cleveland Heights, OH 44118 USA; 2 Future Biologics, Lawrenceville, GA USA; 3 South Texas Orthopedic Research Institute (STORI Inc.), Laredo, TX USA; 4 Veterans in Pain (V.I.P.), Los Angeles, CA USA; 5 Department of Musculoskeletal Disorders, School of Medicine and Surgery, University of Salerno, Fisciano, Italy; 6 San Giovanni di Dio e Ruggi D’Aragona Hospital “Clinica Orthopedica” Department, Hospital of Salerno, Salerno, Italy; 7 Barts and the London School of Medicine and Dentistry, Centre for Sports and Exercise Medicine, Queen Mary University of London, London, UK; 8 School of Pharmacy and Bioengineering, Keele University School of Medicine, Stoke on Trent, UK; 9 School of Osteopathic Medicine, University of The Incarnate Word, San Antonio, TX USA; 10 Future Physicians of South Texas, San Antonio, TX USA; 11 Department of Orthopaedics, School of Medicine, Case Western Reserve University, Cleveland, OH USA; 12 Southern Illinois University, School of Medicine, Springfield, IL USA; 13 Bohlander Stem Cell Research Laboratory, Department of Biology, Bradley University, Peoria, IL USA; 14 Advanced Spine Pain Solutions, Laredo, TX USA; 15 Laredo Sports Medicine Clinic, Laredo, TX USA

**Keywords:** Musculoskeletal injuries, Knee osteoarthritis, Regenerative medicine, Stem cells, Progenitor cells, Growth factors, Cytokines, Extracellular vesicles, Exosomes, Clinical trial

## Abstract

**Background:**

Musculoskeletal conditions are highly prevalent, and knee OA is most common. Current treatment modalities have limitations and either fail to solve the underlying pathophysiology or are highly invasive. To address these limitations, attention has focused on the use of biologics. The efficacy of these devices is attributed to presence of growth factors (GFs), cytokines (CKs), and extracellular vesicles (EVs). With this in mind, we formulated a novel cell-free stem cell-derived extract (CCM) from human progenitor endothelial stem cells (hPESCs). A preliminary study demonstrated the presence of essential components of regenerative medicine, namely GFs, CKs, and EVs, including exosomes, in CCM. The proposed study aims to evaluate the safety and efficacy of intraarticular injection of the novel cell-free stem cell-derived extract (CCM) for the treatment of knee OA.

**Methods and analysis:**

This is a non-randomized, open-label, multi-center, prospective study in which the safety and efficacy of intraarticular CCM in patients suffering from grade II/III knee OA will be evaluated. Up to 20 patients with grade II/III OA who meet the inclusion and exclusion criteria will be consented and screened to recruit 12 patients to receive treatment. The study will be conducted at up to 2 sites within the USA, and the 12 participants will be followed for 24 months. The study participants will be monitored for adverse reactions and assessed using Numeric Pain Rating Scale (NPRS), Patient-Reported Outcomes Measurement Information System (PROMIS) Score, Knee Injury and Osteoarthritis Outcome Score Jr. (KOOS Jr.), 36-ietm short form survey (SF-36), Single Assessment Numeric Evaluation (SANE), physical exams, plain radiography, and magnetic resonance imaging (MRI) with Magnetic Resonance Observation of Cartilage Repair Tissue (MOCART) score for improvements in pain, function, satisfaction, and cartilage regeneration.

**Discussion:**

This prospective study will provide valuable information into the safety and efficacy of intraarticular administration of cell-free stem cell-derived extract (CCM) in patients suffering with grade II/III knee OA. The outcomes from this initial study of novel CCM will lay the foundation for a larger randomized, placebo-controlled, multi-center clinical trial of intraarticular CCM for symptomatic knee OA.

**Trial registration:**

Registered on July 21, 2021. ClinicalTrials.gov NCT04971798

## Background

Osteoarthritis (OA) and other orthopedic acute and degenerative conditions affect millions of people each year, resulting in marked pain and disability [1–3]. Knee OA is the most prevalent and is estimated to affect 67 million people by the year 2030 [4]. Conservative modalities are limited, as they do not reverse the underlying pathology and may only provide symptomatic relief [3–11].

To address the limitations of traditional conservative modalities, there has been substantial interest in biologics for musculoskeletal regenerative medicine applications [6, 12]. The efficacy of biological products is attributed to the presence of growth factors (GFs), cytokines (CKs), and extracellular vesicles (EVs) including exosomes [13–16].

First-generation biologics, specifically whole stem cell products, are not without limitations, including establishing a reliable source with a stable phenotype, genetic instability and chromosomal aberrations, intravenous administration-related toxicities caused by the physical trapping of the cells in the lung microvasculature, rejection by the host, formation of ectopic tissue, and tumorigenicity [17–20].

When considering how to harness the value of current biologics into a next generation product that can address existing limitations, it is important to consider the current knowledge regarding the mechanism of action of stem cell products. The recent literature regarding the beneficial effects of mesenchymal stem cells (MSCs) postulates that the mechanism of action does not result from their ability to grow and differentiate. Rather, it is secondary to their secretion of bioactive molecules such as GFs, CKs, and exosomes [21–24]. GFs, secreted from stem cells, induce signal transduction pathways that initiate cell migration, proliferation, growth, and differentiation [25]. CKs, similarly, can regulate inflammation, immune response, cellular differentiation, and tissue remodeling [26]. Exosomes also are secreted by mesenchymal stem cells and act as a paracrine mediator to target cells, providing a regenerative microenvironment for damaged tissues [23, 27, 28].

As the existing literature establishes that these components of stem cells produce definite regenerative responses, we have accordingly sought to establish whether a sub-cellular approach to biologics can provide similar benefits while avoiding the risk profile, including immunogenicity, infection, and the potential for tumorigenicity, associated with whole stem cell products. Supporting this hypothesis, recent studies have demonstrated that MSCs-derived exosomes can act as a cell-free therapeutic alternative to whole cell therapy with great regenerative potential [29–31]. In addition to the benefits by means of risk elimination, there may be further therapeutic benefits of a cellular derived therapeutic approach. For example, exosomes, given their smaller size, have the potential to migrate to target organs efficiently, without getting trapped in the lung microvasculature [32, 33]. Additionally, a higher concentration of “active ingredients” can be administered directly to the patient, which may induce a greater healing response than possible with whole stem cell therapies.

To meet these goals of improving the risk profile and therapeutic benefit of regenerative medicine, we have formulated a novel cell-free stem cell-derived extract (CCM) from human progenitor endothelial stem cells (hPESCs). Our preliminary results demonstrated the presence of several GFs, anti-inflammatory CKs and EVs including exosomes in this formulation [34]. Functionally, this formulation also significantly enhanced cell proliferation and induced stem cell migration [34].

The goal of this proposed study is to evaluate the safety and efficacy of intraarticular injection of this cell-free stem cell-derived extract formulation for the management of knee OA. We hypothesize that intraarticular administration of this cell-free stem cell-derived extract formulation is safe. We also hypothesize that patients receiving intraarticular injection of this formulation will show an improvement in their overall satisfaction, Numeric Pain Rating Scale (NPRS), Patient-Reported Outcomes Measurement Information System (PROMIS) score, and Knee Injury and Osteoarthritis Outcome Score (KOOS Jr.) over a period of 2 years compared to baseline. We wish to test the null hypothesis that there is no difference between baseline and follow-up visits for the outcome measures considered.

## Methods and analysis

The Standard Protocol Item-Recommendations for Intervention Trials (SPIRIT) criteria were used to report this study protocol [35]. The complete SPIRIT checklist can be found within the supplementary data.

### Study design

**Fig. 1:**
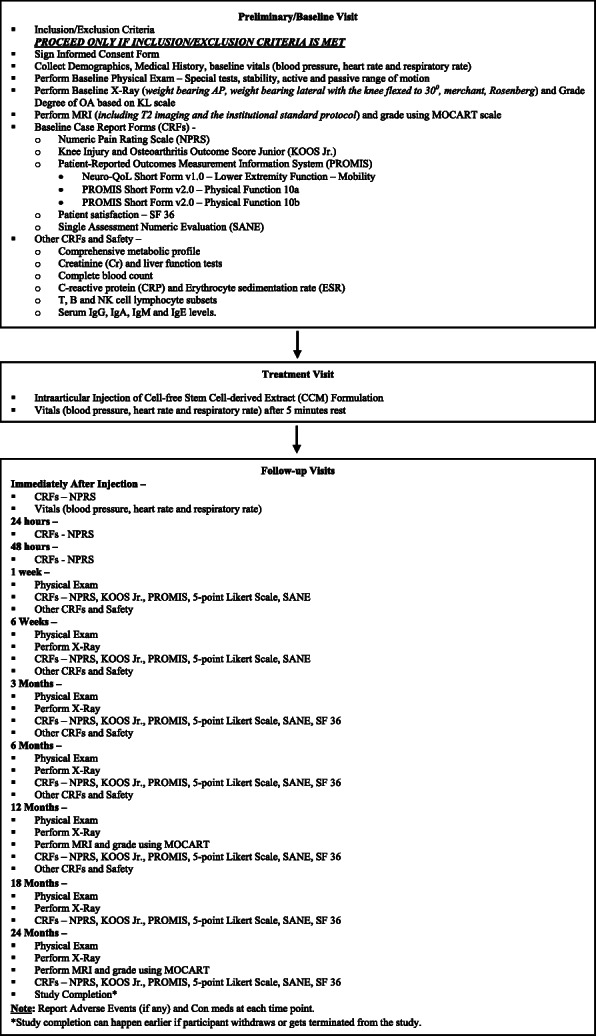
Summary of trial design

**Fig. 2:**
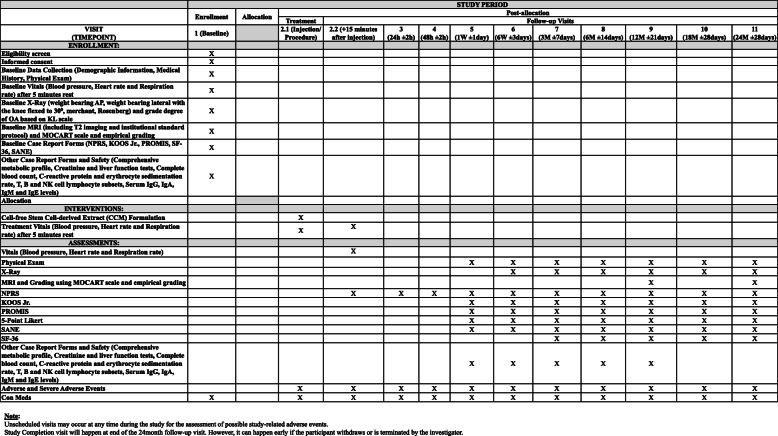
Standard protocol items: recommendations for interventional trials (SPIRIT) flowchart

Up to 20 patients with grade II/III OA who meet the inclusion and exclusion criteria will be consented and screened to allow for approximately 12 patients to receive treatment for this non-randomized, open-label, multi-center, prospective study. This study will be conducted at two sites within the USA, and the participants will be followed for 24 months after the intervention. Figure 1 summarizes the trial design, and Fig. 2 illustrates the enrolment, intervention, and assessment according to the SPIRIT guidelines.

#### Inclusion criteria

Patients will be considered for inclusion in the study if they meet the following criteria:

1. 18 years of age or older
2. BMI of ≤ 35Kg/m^2^
3. Willing and capable of providing informed written consent
4. Willing and capable of subjective evaluation and able to understand written questionnaires
5. Diagnosed with mild to moderate knee OA in only one knee with a grade II/III on the Kellgren Lawrence (KL) grading scale
6. Average knee pain intensity of ≥ 6 on the NPRS
7. Willing to not take any knee symptom modifying drugs through the end of the study
8. Willing and able to comply with study-related requirements, procedures, and visits
9. If female, sexually active, and of childbearing age, patients must be willing to use a reliable form of birth control throughout the duration of the study. If male, sexually active, and with partners of childbearing age, patients must be willing to use contraceptive measures.

#### Exclusion criteria

In addition to the inclusion criteria, patients must not meet any of the following exclusion criteria:

1. Taken any pain medications, including NSAIDS, within 15 days prior to the study injection date.
2. Use of anticoagulants or history of the regular use of anticoagulants
3. History of addiction to dependency producing medications or substance abuse, including alcohol or illicit drugs
4. Mechanical knee symptoms consistent with extensive intraarticular pathology not amenable to injection therapy alone, including clinical or imaging evidence of anterior cruciate ligament, posterior cruciate ligament, medial collateral ligament, lateral collateral ligament, or meniscal pathology
5. Undergone an intraarticular injection of any drug or device including but not limited to corticosteroid, platelet rich plasma or viscosupplementation in the index knee within the last 3 months
6. History of any type of surgery in the index knee
7. Recent (within 3 months) history of traumatic injury
8. Planned elective knee surgery during the duration of the study
9. History of organ or hematologic transplantation
10. History of rheumatoid arthritis or other autoimmune disorders
11. History of immunosuppressive medication/treatment or cancer diagnosis within the last 5 years
12. Current knee infection or history of using antibiotics for a knee infection within the last 3 months
13. Participation within another clinical study or received treatment with any investigational product within 30 days of enrollment
14. Currently pregnant, as determined by urine testing
15. Breastfeeding, or desires pregnancy during the course of the study
16. Contraindications to plain radiography or MRI imaging
17. Diagnosis of progressive neurological disease
18. Diagnosis of an active psychological or psychiatric disorder
19. Pain within other areas and/or medical conditions that could interfere with pain reporting, study procedures, and/or confound evaluation of the study
20. Unresolved major issues of secondary gain (e.g., social, financial, or legal)

### Study intervention

After the patients have meet all inclusion/exclusion criteria during visit 1 (preliminary/baseline), they will receive an intraarticular injection of CCM (2 ml, General Therapeutics LLC, Cleveland Heights, OH, USA) formulation via anterolateral approach by the site principal investigator (PI) utilizing ultrasound guidance per PI’s standard institutional protocol during visit 2.1 (injection visit/procedure).

### Assessment points

The assessments for the study period will begin at visit 1 (preliminary/baseline), where patients will be screened for the inclusion/exclusion criteria and asked to sign the informed consent form. Once enrolled, the demographic information, medical history, medication history, and vitals will be collected. A baseline physical exam (PE) that includes knee ligament laxity and active/passive range of motion evaluation will be performed. Baseline plain radiography (weight bearing anteroposterior, lateral with 30° of knee flexion, Merchant and Rosenberg views) and MRI imaging (including T2 imaging and institutional standard protocol) will be performed for OA grading using the KL scale and Magnetic Resonance Observation of Cartilage repair Tissue (MOCART) score, respectively. Baseline case report forms (CRFs) including NPRS, KOOS Jr., PROMIS, Neuro-QoL Short Form v1.0, Lower extremity function-Mobility, PROMIS Short Form V2.0 - Physical Function 10a and 10b, and patient satisfaction/survey (SF-36 and Single assessment Numeric Evaluation (SANE)) will be collected. Additionally, a comprehensive metabolic profile (CMP), creatinine (Cr), erythrocyte sedimentation rate (ESR), T, B, and NK cell lymphocytes subsets, and serum IgG, IgA, IgM, and IgE levels will be collected. The vitals will be recorded followed by administration of injection by the site PI on visit 2.1. During visit 2.2 (immediately after injection follow-up + 15 min after injection), the vitals and NPRS will be recorded. Visits 3 (24 h follow-up ± 2 h) and 4 (48 h follow-up ±2 h) will be completed via phone interview, and NPRS will be collected. On visit 5 (1 week follow-up ±1 day), a PE will be performed and specific CRFs (NPRS, KOOS Jr., PROMIS, 5 point Likert scale and SANE) as well as a comprehensive metabolic profile (CMP), creatinine (Cr), erythrocyte sedimentation rate (ESR), T, B, and NK cell lymphocytes subsets, and serum IgG, IgA, IgM, and IgE levels will be collected. The exact same process will take place through visits 6 (6 week follow-up ± 3 days), 7 (3 month follow-up ± 7 days), and 8 (6 month follow-up ± 14 days) with a plain radiograph at each visit. SF-36 will also be collected at visits 7 and 8. At visit 9 (12 month follow-up ± 21 days), the participants will undergo the aforementioned processes with an MRI for MOCART grading. At visit 10 (18 month follow-up ± 28 days), the participants will undergo a PE, plain radiography, and CRFs (NPRS, KOOS Jr., PROMIS, 5 point Likert Scale, SANE and SF-36). At visit 11 (24 month follow-up ± 28 days), the participants will undergo the aforesaid processes with an MRI for MOCART grading. An empirical grading form evaluating six distinct articular elements namely, cartilage, osteophytes, subchondral sclerosis, bone marrow lesions, joint effusion, and synovitis, involved in the pathoanatomy of knee osteoarthritis using MRI will also be evaluated at baseline and at 12- and 24-month follow-up visits [36]. Participants may report any adverse events or changes in medications at any point during the study. Unscheduled visits may occur at any time for possible study-related adverse events.

### Endpoints

#### Primary endpoint

1. To determine the safety of intraarticular administered CCM, including monitoring for adverse injection reactions including immunogenic or allergic responses or infection.

#### Secondary endpoints

1. To assess the changes if any in patient-reported outcome measures, NPRS, PROMIS, and KOOS Jr., from baseline to different follow-up time points.
2. To assess cartilage formation via MOCART at 2 years time point and compare it from baseline.
3. To assess patient satisfaction.

### Sample size and statistical analysis

Descriptive statistics will be computed for all study variables. Continuous variables will be described with measures of central tendency (mean, median) and dispersion (range, standard deviation). Categorical variables will be summarized as frequencies and percentages. Comparisons between categorical variables will be compared with the chi-Square test; continuous variables will be compared with Student’s *t* test or nonparametric equivalents. Paired continuous data will be assessed with a paired *t* test or Wilcoxon signed rank test, depending on distribution. Paired categorical data will be assessed with the McNemar’s test. For the longitudinal data, a mixed model repeated measures analysis will be used to examine the between subject factors and the within subject factor of time (baseline, visit 1, visit 2, etc.), as well as their interaction, on the outcome variables of interest. Post hoc tests with corrections for multiple comparisons will be run to determine where significance lies. *P* values < 0.05 will be considered statically significant.

### Data collection and handling

The PI will be responsible for the maintenance of source documents and records for a period of 7 years. Data will be transcribed on paper study CRFs, and the original data will be secured by the PI and made available to the sponsor and study monitors. All CRFs will be subject to initial inspection for omitted data, data inconsistencies, illegible data, and deviations by study monitors. All hard copies of CRFs and media will be stored in a secure location for 7 years.

The PI will be responsible for submitting data and reports as follows:

- AEs: In an ongoing basis. This will be reported in the proper section of the CRF.
- SAEs: Report within 24 h of knowledge of event to sponsor and report to IRB within 5 days as per their regulations.
- Deviations, exceptions, violations of protocol: Report to sponsor within 5 days and report to IRB per their regulations.
- Protocol progress report: Provide a copy to sponsor and IRB as per regulations.
- Study closure report: Provide a copy to sponsor and IRB as per regulations.

### Quality control and assurance

All documents related to the study will be produced and maintained to ensure control and protection of patient’s privacy. The sponsor, study monitors, and representatives of regulatory authorities are permitted to access all study documents (e.g., protocol, CRFs, medical records/files) as needed. All attempts will be made to preserve patients’ privacy and confidentiality.

## Discussion

OA is one of the most common musculoskeletal conditions in the USA, affecting several joints and leading to pain, loss of function, and a decrease in quality of life [37]. This also leads to a substantial burden on the healthcare system [38]. The knee is the most frequently affected joint, and current efforts to mitigate knee OA are focused on decreasing pain, increasing function, and improving quality of life [38]. These current treatment options have limitations, as symptomatic treatment fails to address the underlying pathophysiological processes associated with OA or regenerate injured cartilage [38]. This is one of the several reasons why the field of regenerative medicine and interest in the use of biologics, including cell-free biologics, has increased so rapidly [27, 34, 37].

This clinical trial will be the first to evaluate the safety and efficacy of intraarticular cell-free stem cell-derived extract (CCM) in patients with Kellgren grade II or III knee OA. We anticipate that the intraarticular injection of CCM is safe, and participants will show an improvement in their pain, function, quality of life, and overall satisfaction. We also hypothesize that the cartilage formation over a period of 2 years compared to the baseline visit will improve. The positive outcomes from this trial will also lay the foundation for a large randomized, placebo-controlled, multi-center trial of intraarticular CCM for symptomatic knee OA.

## Data Availability

The datasets used and/or analyzed during the future study will be available from the corresponding author on reasonable request.

## Abbreviations

CCM: Cell-free stem cell-derived extract
CKs: Cytokines
CRFs: Case report forms
EVs: Extracellular vesicles
GFs: Growth factors
hPESCs: Human progenitor endothelial stem cells
KL: Kellgren-Lawrence scale
KOOS Jr.: Knee Injury and Osteoarthritis Outcome Score
MOCART: Magnetic Resonance Observation of Cartilage Repair Tissue
MSCs: Mesenchymal stem cells
NPRS: Numeric pain rating scale
OA: Osteoarthritis
PI: Principal investigator
SANE: Single Assessment Numeric Evaluation
SPIRIT: Standard Protocol Items-Recommendations for Intervention Trials
